# Green Dentistry and Sustainability in Oral Healthcare: A Systematic Review

**DOI:** 10.3390/dj14060377

**Published:** 2026-06-17

**Authors:** Thomas Gerhard Wolf, Linde Müßig, Kerstin Paulmann, Demetrio Lamloum, Guglielmo Campus

**Affiliations:** 1 Department of Restorative, Preventive and Pediatric Dentistry, School of Dental Medicine, University of Bern, CH-2010 Bern, Switzerland; 2 Department of Periodontology and Operative Dentistry, University Medical Center of the Johannes Gutenberg University Mainz, D-55131 Mainz, Germany; 3 Free Association of German Dentists, D-53173 Bonn, Germany; 4 Department of Cariology, Institute of Odontology, Sahlgrenska Academy, University of Gothenburg, SE-41390 Gothenburg, Sweden; 5 Graduate School for Health Sciences, University of Bern, CH-3012 Bern, Switzerland; 6 Section for Oral Health, Heidelberg Institute of Global Health, Faculty of Medicine, Heidelberg University, D-69120 Heidelberg, Germany; 7 University Hospital Heidelberg (UKHD), D-69120 Heidelberg, Germany; 8 Department of Oral and Maxillofacial Sciences, Sapienza University of Rome, 00161 Rome, Italy

**Keywords:** carbon footprint, dental education, dental sustainability, eco-friendly materials, green dentistry, life cycle assessment, sustainable dentistry, sustainable practices

## Abstract

**Background**: This systematic review evaluates the evidence on sustainable practices in dentistry. It focuses on effective measures, innovative technologies, strategies for reducing the carbon footprint, life cycle assessments (LCA), attitudes toward “green” dentistry, and educational approaches. **Methods**: A systematic search was conducted in five databases (Cochrane Library, Embase, LILACS, MEDLINE via PubMed, and Scopus) without language restrictions in accordance with PRISMA. The review was registered in PROSPERO (CRD420251056821). **Results**: A total of 2395 records were identified; after removing 394 duplicates, 2001 remained for screening. After title and abstract screening, 154 full-text articles were evaluated, of which 51 studies were included. The included studies addressed life cycle assessments of dental materials, sustainable clinical practices, and educational measures. Environmentally friendly materials and procedures, such as reusable personal protective equipment and water-saving technologies, demonstrate significant potential for reducing environmental impact. Despite generally high acceptance among dentists and patients, implementation is often limited by financial and knowledge-related barriers. **Conclusions**: The implementation of sustainable materials and procedures is crucial for reducing environmental impact. Equally important are the integration of ecological content into education and appropriate financial and political frameworks to promote sustainable dentistry.

## 1. Introduction

Sustainability has emerged as a critical concern in healthcare, including dentistry, due to its substantial environmental impact. The World Health Organization (WHO) emphasized the need for environmentally sound and minimally invasive oral healthcare in its Global Oral Health Strategy, urging member states to adopt sustainable approaches [1]. Similarly, the United Nations’ 2030 Agenda for Sustainable Development provides a universal framework for integrating environmental, economic, and social sustainability across sectors. Its 17 Sustainable Development Goals (SDGs) are also relevant to oral healthcare [2]. Dental care contributes significantly to greenhouse gas emissions. For example, the National Health Service (NHS) in the UK reports that dental services account for 675 kilotons of CO_2_ equivalents annually, with travel (64.5%), procurement (19%), and energy (15.3%) as major contributors [3]. Common procedures such as dental examinations and scaling have substantial environmental footprints due to their high frequency [3]. In response to these challenges, the FDI World Dental Federation has proposed five strategic goals for sustainable dentistry: reducing the environmental footprint of dental practices, promoting sustainable materials and processes, increasing awareness among stakeholders, encouraging supportive policy development, and fostering innovation and research [4]. These principles serve as a foundation for integrating sustainability into all facets of dental practice. The concept of “green dentistry” promotes technologically advanced, environmentally responsible, and patient-centered dental care that reduces environmental impact while supporting the well-being of patients and professionals [5]. This includes the adoption of reusable personal protective equipment (PPE), eco-friendly dental materials, and efficient waste and water management systems [6,7,8]. Recent studies have explored the environmental benefits of green practices, economic feasibility, and the social dimensions of sustainability in dentistry [6,7,9,10,11]. Life cycle assessment of materials and procedures offer insights into strategies for reducing environmental impact [6,7], while educational interventions and awareness campaigns are essential to fostering pro-environmental behaviors among both dental professionals and patients [9,10,11]. Despite growing evidence, implementation remains limited. Financial constraints, knowledge gaps, and systemic barriers continue to impede progress. This systematic review aims to consolidate current evidence on green dentistry, focusing on effective practices, innovative technologies, and awareness strategies. Following the PRISMA guidelines [12], this review evaluates studies on life cycle assessments, educational approaches, and attitudes toward sustainable dentistry. The findings may help identify feasible pathways to reduce dentistry’s carbon footprint and align dentistry with global sustainability goals.

## 2. Materials and Methods

### 2.1. Search Strategy

A comprehensive and systematic search was conducted in accordance with the PRISMA guidelines [12]. Searches were performed without language restrictions across five electronic databases: Cochrane Library, Embase, LILACS, MEDLINE via PubMed, and Scopus to identify studies relevant to sustainable practices in dentistry. This systematic review was registered in the International Prospective Register of Systematic Reviews (PROSPERO) (CRD420251056821). The PRISMA 2020 checklist is provided as Supplementary Table S1. The search strategy was designed to capture a broad range of studies related to green and eco-friendly dentistry, carbon footprint reduction, and sustainable technologies and practices in the dental field published up to January 2026. To ensure a robust retrieval, the core search terms were combined with database-specific controlled vocabularies, such as Medical Subject Headings (MeSH) in PubMed and adapted to the specific syntax and Boolean operators for each database. The foundation search string used was as follows: “green dentistry” OR “sustainable dentistry” OR “eco-friendly dentistry” OR “green dental technology” OR “green dental environment” OR (“carbon footprint” AND (dental OR dentistry)).

### 2.2. Inclusion and Exclusion Criteria

The review included original studies that addressed sustainable practices, materials, or technologies in dentistry; evaluated the carbon footprint or environmental impact of dental procedures or materials; and focused on attitudes, education, and awareness in green dentistry. Studies that were editorial in nature, such as letters to the editor or review articles without original research, were excluded. In addition, studies that were not available in full text or were not directly related to dental practices or the sustainability of the dental sector were excluded.

### 2.3. Data Extraction and Synthesis

Data were extracted from each included study and then synthesized and grouped into thematic categories for further analysis, allowing a comprehensive view of the current state of sustainability in dentistry across the following categories: Life cycle assessments in green dentistry, sustainability and ecological footprint, attitudes toward green dentistry, education and training in sustainable dentistry, sustainable practices in dentistry, new materials and sustainable technologies, and specific country and cultural analyses.

Due to the considerable heterogeneity of the included studies and the lack of comparable quantitative outcome measures, a meta-analysis was not feasible. Therefore, a narrative synthesis was performed.

### 2.4. Assessment of Study Quality and Risk of Bias

The methodological quality and risk of bias of the included studies were assessed using the adapted assessment tool developed by the National Heart, Lung, and Blood Institute (NHLBI) (www.nhlbi.nih.gov/health-topics/study-quality-assessment-tools; last accessed 16 June 2026) (Table S2). Since many of the included studies were non-interventional and heterogeneous in design, including life cycle assessment, cross-sectional surveys and qualitative approaches, several NHLBI criteria were not directly applicable. In cases of disagreement that could not be resolved by consensus, an additional review was conducted by a third reviewer.

## 3. Results

**Figure 1:**
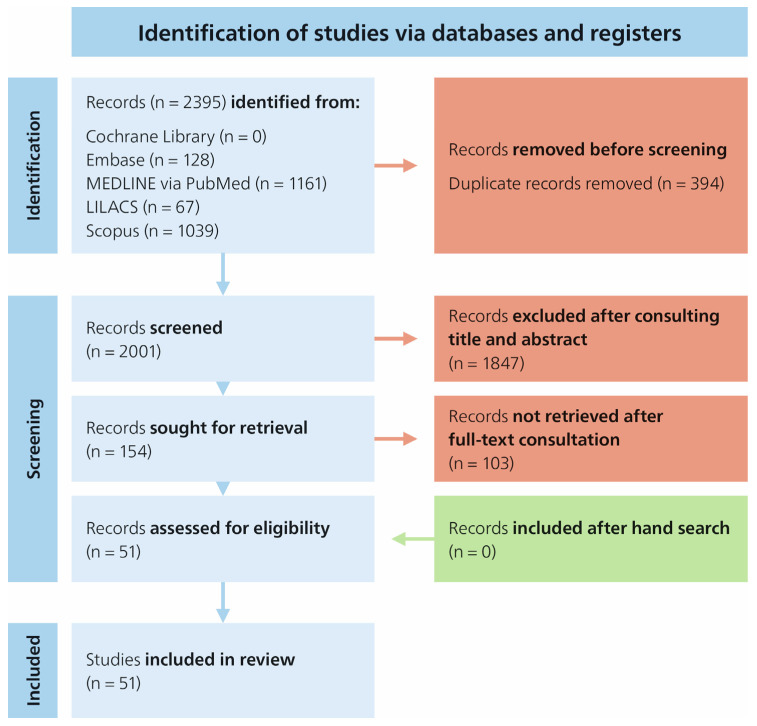
PRISMA-Flowchart.

The current systematic literature search in five electronic databases yielded a total of 2395 articles. After merging the search results from the different databases, 394 duplicates were removed, and 2001 articles were screened based on title and abstract. 1847 articles were excluded after screening title and abstract. Out of the 154 reports requested for retrieval, 51 studies met the inclusion criteria and were included. A total of 103 articles were deemed irrelevant after thorough screening due to an irrelevant focus, inappropriate methodology or unclear study design. A hand search of the bibliographies did not yield any additional references. A total of 51 articles were included in this systematic review. The selection process is illustrated in the PRISMA flow diagram (Figure 1).

**Figure 2:**
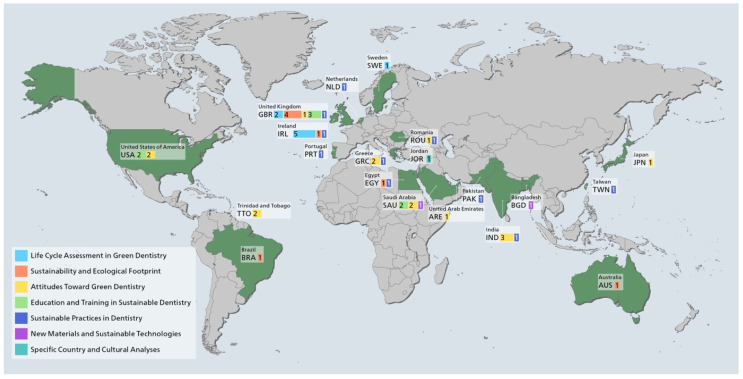
Global distribution of included studies by country and thematic domains: life cycle assessment in green dentistry; sustainability and ecological footprint; attitudes toward green dentistry; education and training in sustainable dentistry; sustainable practices in dentistry; new materials and sustainable technologies; specific country and cultural analyses. The numbers indicate the number of publications in the respective field.

The global distribution of the included studies by country and thematic domain is shown in Figure 2.

### 3.1. Sustainability and Ecological Footprint

Ten LCA studies in dentistry reveal substantial environmental impacts associated with dental practices and materials. Tan et al. [6] found Hawley retainers have a lower long-term impact than Essix retainers, while Duane et al. [13,14,15] emphasized that travel, waste, and procurement are major contributors to the carbon footprint of dental care. They compared their data from 2015 and 2023 (published January 2024) on waste management and water use emissions and found that, despite changes in data collection methods, water use emissions had decreased while waste emissions had increased. Smith et al. [16] showed that dental amalgam has the highest environmental impact among restorative materials. Almutairi et al. [17] highlighted the benefits of reusable over disposable personal protective equipment (PPE). Suresh et al. [7] and Borglin et al. [18] demonstrated that sustainable changes, such as water conservation and eco-friendly materials, greatly reduce the dental footprint. Among fluoride-based preventive measures, water fluoridation has the lowest environmental impact [19]. Studies on national programs and inter dental cleaning aids further stress the importance of adopting greener practices in dentistry [20]. These findings collectively advocate for sustainable practices, materials, and waste management to minimize dentistry’s environmental burden.

Further studies emphasize strategies for reducing the environmental footprint in dentistry. Duane et al. introduced a carbon calculator to help dental practices measure and reduce emissions [15], while their earlier work [3,21] estimated a significant carbon footprint for NHS primary dental care in England, highlighting the need for sustainable practices. The ECODENT model offered by another study encourages pro-environmental behaviors among dentists to foster sustainability [22]. Lyne et al. [23] emphasized that fluoride varnish application within routine or school-based programs minimizes environmental impact, while Martin et al. [10] revealed that patients with higher dental needs contribute to greater carbon emissions and waste. Oral healthcare is less carbon intensive than restorative dental treatment. Promoting oral hygiene and reducing caries reduces high-carbon-intensive dental treatment [24]. Byrne et al. [25] found with a LCA that reusable stainless steel examination kits are more sustainable than disposable plastic alternatives. De Bortoli et al. [26] highlighted that ceramic materials in implant dentistry are more eco-friendly than metals, suggesting a shift toward metal-free practices. Elwan et al. [27] showed that Egyptian dental clinics produce a substantial carbon footprint, predominantly due to patient travel, with staff travel and electricity consumption also significant factors; further impacted by the absence of recycling and depreciation costs. Finally, research from Scotland demonstrated that strategic changes in dental practices can contribute to a low-carbon future. Interestingly, findings indicate that older clinics may have a lower carbon footprint compared to newer facilities [21].

### 3.2. Attitudes Toward Green Dentistry

The 14 studies in different countries on attitudes toward green dentistry show generally positive perceptions but varying levels of implementation. Hoyte et al. [11], Carr et al. [28], Haque et al. [29,30], and Prathima et al. [31] demonstrated that patients in Trinidad and Tobago, Saudi Arabia and India are willing to accept longer appointments and higher costs for eco-friendly practices but are less willing to compromise on health and aesthetics [32]. Dental professionals and students across different regions, including Greece [33], the UAE [9], India [34] and the USA [35], expressed support for sustainability in dentistry, although knowledge gaps and financial constraints were identified as barriers [36,37]. Word of Mouth communication may encourage dentists to adopt pro-environmental behaviors in a shorter time [22]. In Japan health consciousness was positively associated with recycling behavior, but there was no significant association with green purchasing behavior [38].

### 3.3. Education and Training in Sustainable Dentistry

Seven studies on integrating environmental sustainability into dental education reflect the growing importance of embedding sustainability across curricula. Dixon et al. [39] developed strategies to embed sustainability into the dental curriculum. Leung et al. [40] demonstrated that educational interventions increase students’ knowledge and foster sustainable behaviors in clinical practice.

In 2024, Durnall et al. [41] found that 97% of students expected more responsibility in dentistry but currently have little exposure to ESD (Education for Sustainable Development) in their undergraduate studies. Compared to disposable instruments, reusable instruments require only 37% of the water and produce only 36% of the waste [42]. A study in Bucharest [43] highlighted that proper education and awareness are crucial for promoting sustainable practices. Overall, the need to integrate sustainability into the dental curriculum and to provide ongoing education to advance sustainable practices remains key, as shown by Al-Qarni et al. [44] and Jamal et al. [45].

### 3.4. Sustainable Practices in Dentistry

The nine studies on sustainable practices in dentistry highlight both progress and challenges. Veress et al. [46] found that while dentists are open to eco-friendly practices, many need practical guidance. Soliman et al. [8] emphasized the water-saving potential of green dental technologies. Volgenant et al. [47] and Neves et al. [48] identified infection control and costs as significant barriers, despite strong sustainability adoption in some clinics. Younger professionals and postgraduates demonstrated a better understanding of sustainable waste management practices [49,50,51]. Hsu et al. [52] highlighted the importance of patient-centered design in promoting sustainable practices, while Grose et al. [53] advocated for staff engagement.

### 3.5. New Materials and Sustainable Technologies

Two studies on new materials and sustainable technologies in dentistry highlight promising eco-friendly innovations. Chowdhury et al. [54] demonstrated the green synthesis of zirconium nanoparticles using ginger and garlic, showing strong antimicrobial properties for dental implants. El-Rab et al. [55] investigated copper nanoparticles synthesized from Cupressus extract, which exhibited potent antibacterial effects against periodontal bacteria, especially when combined with clindamycin. Additionally, further research into the sustainability of different fluoride delivery methods revealed opportunities to reduce environmental impact [19].

### 3.6. Specific Country and Cultural Analyses

Regional differences in sustainable dentistry practices were highlighted across four studies. Haque et al. [29] surveyed Saudi dental students, revealing moderate awareness of Sustainable Development Goals (SDGs) but high awareness of sustainable dental practices (SDPs). The study also identified positive attitudes toward sustainable dentistry in the general Saudi population, with participants willing to make compromises for environmentally friendly practices. Gender influenced pro-environmental behaviors, and clinical exposure was associated with increased awareness. Al-Qarni [44] found significant improvements in knowledge about green dentistry among Saudi dental faculty and students after an educational intervention, emphasizing the need for curriculum integration. Al Shatrat et al. [37] revealed that while Jordanian dentists have strong knowledge of environmentally friendly dental practices, implementation remains limited due to financial constraints and a lack of government incentives. The results are depicted in Table 1.

## 4. Discussion

The objective of this systematic review was to identify existing knowledge about sustainability in dentistry, focusing on effective practices, cutting-edge technologies, and awareness strategies to reduce the carbon footprint in the dental sector. The findings reveal a range of strategies for improving sustainability within dentistry, from material choices and clinical practices to shifting attitudes toward environmental responsibility. These findings illuminate both the challenges and the opportunities for advancing sustainability in the field.

The results of LCA regarding dental materials align with prior research, particularly in highlighting the environmental impact of materials like dental amalgam and resin-based composites (RBC). Smith [16] and Tan et al. [6] both support the call for replacing high-impact materials like amalgam, RBC and Invisalign aligners with more sustainable alternatives, such as retainers with a longer lifespan [6]. Similarly, the study by Almutairi et al. [17], which emphasizes reusable PPE, aligns with broader healthcare literature advocating for reducing single-use plastic. Studies like Duane et al. [15], which introduced carbon calculators to track emissions, support the growing consensus around the need for more sustainable procurement and operational strategies in dental practices. This is further reflected in findings by Byrne et al. [25] and Abed et al. [20], which advocate for transitioning to reusable and eco-friendly dental tools, such as stainless-steel kits and bamboo floss. Attitudinal studies emphasize a notable “knowledge-action gap” in sustainable dentistry. Despite widespread support for sustainability, actual practice remains hindered by financial constraints, knowledge gaps, and perceived costs [36,37]. Educational interventions, as highlighted by Leung et al. [40] and recommended in a framework proposed by Duane et al. [55], offer a solution by integrating sustainability into dental curricula and fostering environmental responsibility among professionals and students.

Several studies have identified staff commutes and patient travel as significant contributors to the carbon footprint of dental care. This suggests that measures such as consolidating appointments into longer sessions and promoting preventive programs in schools could help reduce environmental impact [14,27].

Future research should focus on developing water-saving processes [8] more environmentally sustainable alternatives to resin-based composite (RBC), environmentally sustainable alternatives to antibiotics [55] and green-synthesized zirconium nanoparticles [54]. In addition, the development of ways to reduce detergents, soap and surface disinfectants may help reduce environmental harm [18].

When implementing greater sustainability in dental practices, infection control often conflicts with measures such as reducing chemical use. Nevertheless, general measures are highly feasible.

One of the first steps should be raising environmental awareness among dentists and dental teams [39,41,45]. Within daily operations, energy consumption can be reduced by switching to LED lighting and utilizing modern, power-saving equipment [3]. Furthermore, more eco-friendly commuting can be encouraged through carpooling, public transit, cycling to work, or consolidating patient appointments. For dental laboratory work, it is also beneficial to rely on regional providers [3,15,27].

Transitioning to digital radiography can substantially reduce the use of chemical processing agents and associated waste streams [44]. Paper waste and impression material can be minimized through comprehensive digitalization [7]. Plastic cups and disposable patient drapes can be replaced with paper alternatives, or patients might bring their own cups and towels from home. Whenever hygienically permissible, reusable instruments should be preferred [13,17,25].

Dentists can pay attention to sustainability-certified workwear [13,18,24]. Expanding the concept of sustainability in oral healthcare requires moving beyond environmentally conscious waste management and resource efficiency toward strategies that promote long-term oral health and clinical longevity. In this context, minimally invasive dentistry and preventive approaches, such as the use of biomimetic hydroxyapatite to enhance enamel remineralization and early periodontal interventions may help reduce the need for resource-intensive restorative procedures over time [57,58]. Furthermore, greater emphasis on patient education and preventive care could contribute to maintaining oral health, thereby decreasing the demand for extensive and repeated treatments throughout a patient’s lifetime [10,24].

A key strength of this systematic review lies in its comprehensive approach, encompassing life cycle assessments, attitudes toward sustainability, and educational interventions. The review’s inclusion of cross-regional studies also enhances the global relevance of its findings, presenting a broad perspective on sustainable dentistry. However, limitations exist. Most studies in this review come from high-income countries, limiting generalizability to low- and middle-income settings where different sustainability challenges may prevail. Furthermore, while our search strategy combined comprehensive terms for green dentistry and carbon foot printing with controlled vocabularies (MeSH), it focused on literature explicitly framed around sustainability. Studies addressing related sub-topics might not have been fully captured. Beyond this, the compiled evidence is limited by high methodological heterogeneity and varying quality. Many included studies utilize cross-sectional surveys prone to bias. Consequently, despite the growing interest in sustainable dentistry, the overall strength of evidence remains limited, warranting caution in generalizing findings. Additionally, reliance on self-reported data in some attitudinal studies introduces the risk of bias. The lack of long-term follow-up data also restricts the ability to assess the durability of sustainable practices and technologies.

The findings of this review, particularly those concerning material assessments and procedural changes, are broadly applicable to dental practices worldwide. However, generalizability is more complex regarding attitudes and implementation, which can vary widely depending on local socioeconomic conditions, cultural factors, and policy frameworks. While the global trend toward sustainability is clear, the specific strategies for implementation may require regional adaptation.

## 5. Conclusions

This systematic review indicates that, like many other areas of healthcare and daily life, dentistry is associated with significant environmental impacts. Travel, procurement and single-use materials appear to account for the largest share of dentistry’s ecological footprint [3,13,15,17,25,27]. Current life cycle assessments suggest that the use of reusable protective equipment, stainless steel sets, ceramic materials and water-saving technologies have the potential to significantly reduce emissions. Although tools such as carbon calculators [15] are valuable management approaches, they have mainly been applied in individual countries so far. Studies indicate that, while patients, students and dentists generally support sustainable dentistry, financial, aesthetic and knowledge-based barriers still impede widespread implementation [36,37]. While educational initiatives show positive tendencies, sustainability is still not sufficiently embedded in curricula [39,40,41]. Initial research into environmentally friendly nanomaterials and technologies is promising but requires reliable long-term data [54,55]. Overall, the available evidence suggests that sustainable dentistry is most likely to be achieved through a combination of environmentally friendly materials, education, changed practice procedures, and political and economic incentives. While current literature highlights the ecological potential of these strategies, transitioning them into routine practice requires addressing existing gaps regarding their long-term clinical efficacy and financial viability. Consequently, future research agendas should expand beyond standardized international life cycle analyses and cross-cultural comparisons. Specific focus should be placed on the development of materials with a lower environmental impact, the conduct of comprehensive cost–benefit assessments, and the establishment of implementation science frameworks for longitudinal evaluations within real-world clinical environments.

## Figures and Tables

**Table 1:** Synthesis of included studies, grouped by thematic domains: life cycle assessment in green dentistry; sustainability and ecological footprint; attitudes toward green dentistry; education and training in sustainable dentistry; sustainable practices in dentistry; new materials and sustainable technologies; specific country and cultural analyses. The columns indicate author(s) (year), quality assessment, location, study type, approaches, and main findings. Abbreviations used in the study type column: CER, carbon emission reduction; CS, comparative study; CSS, cross-sectional survey; DORS, design-oriented research study; ESD, experimental study design; IAS, impact assessment study; LCA, life cycle assessment; MD + ES, model development and evaluation study; PSS, pilot survey study; QS, qualitative study; and TDS, tool development study.

Author(s) (Year)	Quality Assessment	Location	Study Type	Approaches	Main Findings
**Sustainability and Ecological Footprint**					
Duane et al. (2024) [14]	Poor	IRL	LCA	Calculating the carbon footprint of dental practice, measuring greenhouse gas emissions associated with all stages of a product or service’s life	Staff and patient travel accounted for 61% of the total footprint; waste’s contribution increased from 0.3% to 1%, while the contribution from water usage decreased
Suresh et al. (2024) [7]	Fair	IRL	LCA	Seven functional units were compared: Emailing, paper use, toilet water, dishwasher, clinical water, printing and photocopying, reusable/disposable syringe tips	Green Impact Toolkit in dental practices can significantly reduce the environmental impact, especially in areas of procurement, waste management, and water use
Tan et al. (2024) [6]	Poor	IRL	LCA	Sixteen impact categories were assessed for Hawley and Essix Retainers, including transport, lifespan, manufacturing, disposal and production	Hawley retainers have a higher manufacturing impact but are more sustainable due to their longer lifespan
Abed et al. (2023) [20]	Fair	GBR	LCA	Comparing different items for daily interdental cleaning, depending on the environmental impact category	The smallest environmental footprint came from bamboo floss (one impact category) followed by regular floss and sponge floss (three impact categories)
Smith et al. (2023) [16]	Poor	GBR	LCA	Dental restorative materials were assessed: dental amalgam, resin-based composite (RBC) and glass polyalkenoate cements (GIC)	Main sources of environmental impacts were: Amalgam: from material use; RBC: from energy use in processing and packaging; and GIC: from material and packaging energy
Almutairi et al. (2022) [17]	Fair	IRL	LCA	Comparing different types of personal protective equipment (PPE) used in dental care during the COVID-19 pandemic.	In all categories, disposable materials have a higher environmental impact than reusable ones
Byrne et al. (2022) [25]	Poor	IRL	LCA	Assessment of the environmental impact of reusable and disposable dental examination kits	Single-use examination kits made of plastic cause significantly greater environmental and human health impacts than reusable stainless-steel kits
Duane et al. (2022) [19]	Fair	IRL	LCA	Quantifying the environmental impact of water fluoridation compared to part one and part two	Community water fluoridation has the lowest environmental impact (EI) compared to other fluoride-based prevention methods like toothpaste, mouth rinses, and varnishes
Borglin et al. (2021) [18]	Poor	SWE	LCA	A dental examination in a hypothetical dental practice	The largest impacts were found in freshwater eutrophication, and human toxicity (cancer effects); small changes, like using sustainable materials for clothing and more eco-friendly soaps and detergents, could reduce the environmental burden
Duane et al. (2020) [13]	Fair	IRL	LCA	Analyzing contributors of a root canal treatment (RCT)	Major contributors include dental clothing, disinfectants, disposable bibs, single-use instruments, and electricity; reducing impact may involve using eco-friendly materials and single-visit treatments
Elwan et al. (2025) [27]	Good	EGY	ARS, CER	Data collection by interviews on various aspects including patient visits, staff numbers, energy consumption, waste, water usage, and travel patterns of patients and staff	Patient travel was the main reason for the largest portion of the footprint (46%), followed by staff travel (20%), and energy consumption (18%)
Martin et al. (2024) [10]	Fair	GBR	ES	CO_2_ and SUP (single-use plastics) waste was calculated for five patient categories with common preventable diseases	The most effective way to reduce environmental impact is by promoting preventive oral healthcare
Sowa et al. (2023) [42]	Fair	AUS	CCA	Students’ and faculty’s perception regarding environmental sustainability in dentistry (ESD), reusable instruments (RI), disposable instruments (DI)	In terms of environmental impacts, the use of instruments based on current practice required 37% of water and energy use of an RI-only alternative and generated 36% waste of the DI-only alternative
Lyne et al. (2022) [23]	Fair	GBR	LCA	Quantifying the environmental impact of a five-year-old child receiving two fluoride varnish (FV) applications	Integrating FV with other services and using reusable equipment in schools can reduce environmental burden
Bowden et al. (2021) [24]	Poor	GBR	IAS	Comparing the carbon footprint of dental treatment with preventive programs	Oral care is less carbon-intensive than restorative dental treatment; promoting oral hygiene and reducing caries, reduces high carbon-intensive dental treatment
De Bortoli et al. (2019) [26]	Fair	BRA	CS	Evaluation of the ecological footprint of various biomaterials used in implant dentistry, comparing traditional metal-based implants with metal-free alternatives	The primary production of ceramic materials tends to have less significant impacts compared to metallic materials, with lower values for the generation of greenhouse gas emissions, water consumption and embodied energy
Duane et al. (2017) [3]	Fair	IRL	QS	Calculates the carbon emissions of NHS primary dental care in England	The highest contributors to emissions were travel 65%, followed by procurement 19%, energy use 15%; examinations accounted for 27% due to their high volume, amalgam/composite restorations contributed 19%.
Duane et al. (2012) [21]	Fair	GBR	ARS, CER	Carbon accounting for indirect and direct emissions; energy and water; waste collection contracts; staff and patient surveys about travel	Older clinics had lower carbon footprints than newer ones due to lower energy consumption; carbon accounting might help to reduce the carbon footprint
**Attitudes Toward Green Dentistry**					
Carr et al. (2024) [28]	Fair	TTO	CSS	Questions concerning attitude towards sustainable dentistry: Willingness to have a longer appointment, willingness to accept alternatives (e.g., pay more, aesthetics, dental health)	Adults in Trinidad and Tobago hold positive attitudes toward sustainable dentistry and are moderately willing to accept trade-offs, especially longer appointment times
Haque et al. (2024) [29]	Fair	SAU	CSS	Knowledge and awareness; a self-administered online questionnaire was distributed to adults aged between 18 and 35 years of both genders	Dental students have a fair understanding of the ESD; influencing factors: Gender and clinical experience influenced the attitude to ESD
Haque et al. (2024) [30]	Poor	SAU	CSS	Online cross-sectional observational survey adults aged 18 years and older, of both genders	Positive attitude towards sustainable dentistry and general willingness to make compromises such as sacrificing time, convenience, costs, and even aesthetics to reduce the environmental impact of dental procedures
Hoyte et al. (2024) [11]	Poor	TTO	CSS	Questionnaire assessed attitude towards and willingness to accept alternatives, which decrease the effect of dental treatment on the environment	Overall positive attitude towards sustainable dentistry and was willing to accept alternatives so that their dental treatment would have less impact on the environment
Nassar et al. (2024) [9]	Fair	ARE	CSS	Measured awareness, attitudes, and practices regarding environmental sustainability in dentistry among dental students	Barriers include lack of knowledge, educational resources, and curriculum space; poor awareness of ESD, but positive attitudes to integrate ESD into the dental curricula
Antoniadou et al. (2023) [33]	Fair	GRC	CSS	Survey about socio-environmental elements, educational elements, resources elements, law and capacity elements	Dental students and dentists exhibit a lack of knowledge regarding the circular economy, EU regulations on mercury disposal, and plastic recycling; recycling at home is more common than at work
Antoniadou et al. (2023) [22]	Fair	GRC	MD+ES	Assessing the impact on dentists’ pro-environmental behaviors through surveys and observational data implementing the ECODENT model	Increasing word-of-mouth parameters, government economic support or training hours while reducing investment and operating costs will make changes in the environmentally friendly behavior of dentists more quickly noticeable
Țâncu et al. (2023) [43]	Fair	ROU	PSS	Questions about awareness of sustainability in dentistry, negative impact of dental activities on environment, approaches for integrating sustainability, COVID-19 impact	Overall, awareness and knowledge about sustainability in dentistry is only moderate, both in terms of sustainable practices and the negative environmental impacts of dental activities
Thakar et al. (2023) [36]	Good	IND	CSS	Survey with 22 questions to determine the dental professionals’ and dental students’ awareness, knowledge, and restrictions to practicing green dentistry	Participants (52%) were not aware of the concept of green dentistry; practitioners of the age group 20–30 years are more willing to transform their dental practice into green practice to reduce the carbon footprint of their clinic
Verma et al. (2020) [34]	Fair	IND	CSS	Assess the knowledge and practice of dental professionals regarding eco-friendly dentistry	The lack of knowledge was identified as the main barrier to adopting green dentistry practices, with many participants unaware of the environmental harm caused by traditional dental practices such as the improper disposal of mercury
Baird et al. (2022) [32]	Fair	GBR	CSS	A questionnaire considering the public’s attitudes towards sustainability in dentistry	Participants generally have positive attitudes towards sustainable dentistry; willingness to compromise varies depending on the trade-off; more willingness to make sacrifices in terms of time, convenience and cost, less willingness to compromise their health or the aesthetics
Gershberg et al. (2022) [35]	Good	USA	CSS	Questionnaire about travel, equipment and supply, energy, waste management, biodiversity and green space, incorporating sustainability into current dental practice, amalgam disposal	The study highlighted a gap in the curriculum and a strong student demand for more sustainability education
Shimoda et al. (2020) [38]	Fair	JPN	CSS	Health consciousness and pro-environmental behavior among health professionals in Japan	Health consciousness is positively associated with recycling behavior, but there is no significant association with green purchasing; the intention to engage in pro-environmental behavior is significantly linked to green purchasing, but not to recycling
Prathima et al. (2016) [31]	Fair	IND	CSS	Knowledge, attitude and practices regarding eco-friendly dentistry	Only 13% of participants were aware of the Eco-Friendly Dentistry (EFD) Association, 76% acknowledged the environmental harm caused by dental practices; 58% reported following proper waste disposal protocols
**Education and Training in Sustainable Dentistry**					
Dixon et al. (2024) [39]	Good	GBR	Mixed	Identification of core content	1. Normalizing the topic, 2. Baseline knowledge, practical application, 3. View and modify teaching, 4. ES transcends all disciplines in dentistry, 5. Safeguarding against misinformation and disinformation
Durnall et al. (2024) [41]	Fair	GBR	CSS	A multi-center, cross-sectional online survey of undergraduate BDS and DH&DT students was carried out using a questionnaire that was distributed to all UK-based dental schools	Regardless their stage of progression, students showed the same interest; 77.9% were concerned, 17.5% very concerned in reducing their own impact, but only 54% try to reduce it
Jamal et al. (2023) [45]	Fair	SAU	CSS	Students’ and faculty’s perception regarding environmental sustainability in dentistry (ESD)	ESD is not sufficiently integrated into dental curricula; most students (87%) and faculty members (83%) agree on the necessity of teaching it; more than 80% demonstrated a lack of knowledge regarding ESD
Leung et al. (2022) [40]	Good	USA	CSS	Raise awareness and knowledge of ESD (environmentally sustainable dentistry) among dental hygiene students	By increasing knowledge, positive attitudes were increased too; cost was the most frequently cited challenge, though students found that the long-term benefits outweighed the initial expenses
Duane et al. (2021) [56]	Good	GBR		Scoping questionnaire on current practice and understanding	Institutions still lack standardized teaching materials
Al-Qarni et al. (2016) [44]	Fair	SAU	CSS	Questionnaire: knowledge about eco-friendly dentistry; eco-friendly strategies such as using digital X-rays, reducing energy consumption, and waste recycling	There is need to incorporate eco-friendly practices into dental education; key barriers to implementation are economic concerns and the initial costs of adopting green practices
**Sustainable Practices in Dentistry**					
Duane et al. (2024) [15]	Fair	IRL	TDS	A specialized carbon calculator for dental practices to compute and monitor their carbon footprints (CFPs)The calculator is freely downloadable and part of a broader ‘green dentistry’ initiative	By raising awareness of their CFP, it encourages progress in ‘green dentistry’ and promotes environmental responsibility in oral healthcare; continuous carbon emission measurement is crucial for a sustainable future
Soliman et al. (2023) [8]	Fair	EGY	Case	Water-saving practices were observed, case study approach of four eco-friendly dental clinics	Water saving potential: Eco-friendly dental technologies, such as dry dental vacuum systems, fast cycle washers, and ECO sterilizers, significantly reduce water consumption in dental facilities
Spaveras et al. (2023) [49]	Fair	GRC	CSS	Questions about dental amalgam and its environmental impact, group of students and group of dentists	Environmental impact of amalgam is considered only moderate; assessments vary considerably depending on professional experience, year of study, and geographical location
Veress et al. (2023) [46]	Fair	ROU	CSS	50 questions about sustainability in dentistry, 98 Responses including travel, number of treatments, location of dental lab, lighting, paper waste, supply order	75% liked the idea of an environmentally friendly dental practice and 99% would take some steps towards environmental awareness
Kamran et al. (2022) [50]	Fair	PAK	CSS	Questions including awareness of Pakistan’s biomedical waste management laws, duration of waste keeping, dental waste transportation regulatory body	Most dental practitioners (98%) and dental students (95%) demonstrated awareness of the health risks posed by biomedical waste; the knowledge of different biomedical waste categories was insufficient
Neves et al. (2022) [48]	Fair	PRT	CSS	The questionnaire collected information about the implementation of environmental sustainability practices in dental clinics within six categories of management	Most respondents (96%) acknowledged the importance of sustainability practices, with the main barriers being costs (45%) and lack of training/information (1%)
Volgenant et al. (2022) [47]	Fair	NLD	QS	Interviews including structural level, dental practice level (training, adoption, implementation, practice improvement), oral health care practitioner level, method and product level	Growing awareness, especially among women and younger professionals; interest in sustainable practices like reducing energy use; high costs and limited decision-making power in larger practices impede implementation
Hsu et al. (2019) [52]	Poor	TWN	DORS	How dental healthscape elements influence (design, ambience, social interaction) clients’ positive emotions, their revisit, based on the stimulus-organism-response model	Positive emotions, such as feeling thoughtful, hopeful, and comfortable, increased clients’ willingness to revisit dental practices; designing the dental environment to foster these emotions is crucial for sustainable dentistry.
Puri et al. (2019) [51]	Poor	IND	CSS	Questions including dental and biomedical waste disposal	Gaps in knowledge were found in understanding waste categories, disposal practices, and eco-friendly techniques
Grose et al. (2018) [53]	Good	GBR	ARS	Interventions: Glove use, waste segregation, procurement of products, energy saving, environmental awareness, sustainability integral to the practice, changes in other aspects of their lives	The Intervention with the action study worked; involving the staff in developing sustainability gave them a sense of community, which increased environmental awareness and positive behavior
**New Materials and Sustainable Technologies**					
Chowdhury et al. (2023) [54]	Good	BGD	ESD	Synthesizing zirconium nanoparticles from garlic and ginger, using eco-friendly processes, followed analyzing their properties and suitability for dental implant applications	Excellent antimicrobial properties; all nanoparticles exhibited antibacterial activity against *S. aureus*, inhibited bacterial adhesion and biofilm formation.
El-Rab et al. (2021) [55]	Good	SAU	ESD	Synthesis of copper nanoparticles from cupressus macrocarpa extract using eco-friendly methods	Green-synthesized CuNPs has the potential as an eco-friendly and cost-effective strategy for combating dental infections; reducing antibiotics as an environmental factor
**Specific Country and Cultural Analyses**					
Al Shatrat et al. (2013) [37]	Fair	JOR	CSS	Knowledge and implementation of eco-friendly dental office strategies	It exists a high level of knowledge about eco-friendly strategies; cost and the lack of government incentives are identified as barriers; there is need for education to promote eco-friendly practices in dentistry

## Data Availability

No new data were created or analyzed in this study. Data sharing is not applicable to this article.

## Supplementary Materials

The following supporting information can be downloaded at: https://www.mdpi.com/article/10.3390/dj14060377/s1, Table S1: PRISMA 2020 checklist [59]; Table S2: Quality assessment tool evaluation.

## Abbreviations

The following abbreviations are used in this manuscript:

CFP	Carbon footprint
CO_2_	Carbon dioxide
CuNP	Copper nanoparticles
DI	Disposable instruments
EI	Environmental impact
EFD	Eco-Friendly Dentistry
ESD	Environmental sustainability in dentistry
FDI	FDI World Dental Federation
FV	Fluoride varnish
GIC	Glass polyalkenoate cements
LCA	Life cycle assessment
LILACS	Latin American and Caribbean Health Sciences Literature
NHS	National Health Service
PPE	Personal protective equipment
PRISMA	Preferred Reporting Items for Systematic Reviews and Meta-Analyses
RBC	Resin-based composite
RCT	Root canal treatment
RI	Reusable instruments
SDG	Sustainable Development Goals (SDGs)
SUP	Single use plastics
UK	United Kingdom of Great Britian and Norther Ireland
WHO	World Health Organization
